# Heterogeneity and cell fate flux in single human pancreatic islet cells

**DOI:** 10.1038/s41419-018-0269-7

**Published:** 2018-02-14

**Authors:** Natasha H. J. Ng, Adrian K. K. Teo

**Affiliations:** 1 Stem Cells and Diabetes Laboratory, Institute of Molecular and Cell Biology, A*STAR, Singapore, Singapore; 2 School of Biological Sciences, Nanyang Technological University, Singapore, Singapore; 3 Department of Biochemistry, Yong Loo Lin School of Medicine, National University of Singapore, Singapore, Singapore; 4 Lee Kong Chian School of Medicine, Nanyang Technological University, Singapore, Singapore

The islets of Langerhans, which form the endocrine compartment of the pancreas, comprise a mixture of distinct hormone-expressing cell populations each of which plays a different functional role, but acts in concert to achieve glucose homeostasis. These cells include β, α, δ, γ (or PP), and ε cells, primarily characterized by their secretion of insulin (INS), glucagon (GCG), somatostatin (SST), pancreatic polypeptide (PPY), and ghrelin (GHR), respectively. As islet dysfunction is a major contributor to diabetes pathogenesis, understanding the unique roles of individual islet cell types is fundamental to understanding the development and progression of diabetes. Distinct differences in islet composition and architecture between rodents and humans have also been widely recognized; hence, efforts are increasingly geared toward studying human pancreatic islet cells.

Human islet expression profiling studies have seen a shift from analyses of whole islets to sorted cell populations using FACS. With the advancement of novel, high-throughput single-cell technologies in recent years, researchers are now able to characterize individual pancreatic cells of an islet on a single-cell basis and this has opened up new perspectives not only in relation to islet composition as a whole but also the heterogeneity and plasticity that exists within each cell population. As opposed to bulk analyses whereby less abundant cell types or cells undergoing transient states are often missed, we now have the potential to identify rare cell types and study their features using single-cell platforms.

In a nutshell, single-cell “-omics” enable clustering of cells based on their expression profile (RNA or protein level) at scale and, therefore, determination of population subtypes based on cell-type-specific markers^1^. A series of seminal studies have analyzed the transcriptomes of human islet cells by single-cell RNA sequencing (RNA-Seq) to characterize the cells with greater resolution and identify cell-type-specific expression signatures^2–8^. Some, but not all, of these studies described the heterogeneity present among each cell type as demonstrated by the distinct subpopulations within the α and β cell populations that arose from differences in expression patterns of maturation markers, proliferative markers, and/or stress genes^3,5,6,9,10^.

Of particular interest were findings by Wang *et al*. acknowledging the presence of rare single cells with “conflicted” α/β signatures that potentially represent transitional or progenitor-like states^6^. The biological significance of single cells with mixed gene expression profiles, however, remains to be determined as the cells were dropped from in-depth analysis to prevent inclusion of potential cell doublets. While comparing between islets from adult and juvenile donors, the authors also reported that pediatric endocrine cells exhibit different α and β cell transcriptome profiles from those of adult cells, indicating an immature differentiation state^6^. The presence of cells with mixed profiles has not been sufficiently addressed in other data sets, as a major limitation in current single-cell RNA-Seq studies is the exclusion of cells that exhibit mixed or ambiguous identities, which were often attributed to contamination or capturing of doublet cells^5–8^. The potential bias as a result of this filtering process limits thorough appreciation of cells at a transitional state that do not necessarily conform to well-established gene expression signatures. Moreover, the trade-off between sequencing a large number of cells and sequencing depth may affect transcript representation within each individual cell, especially for that of low-abundance transcripts.

To circumvent some of these issues, Teo *et al*. conducted a study, published recently in *Cell Death Discovery*, based on an analysis of up to 281 single cells across six adult human islet preparations from healthy donors^11^. Using single-cell quantitative polymerase chain reaction-based gene expression analysis and immunohistochemical approaches, the authors found that, although ~63% of the islet cell population expressed insulin, almost half of all islet cells analyzed were multihormonal, many of which were co-expressing INS and SST or PPY. These multihormonal signatures were supported by the observation that a substantial proportion of INS-positive islet cells exhibited signs of loss of β cell identity because of low expression of key markers of the β-cell functional machinery such as *GCK* and *PCSK2*. In fact, only a small proportion of the INS-positive cells co-expressed the β-cell fate-determining transcription factors *PDX1*, *NKX6.1,* and *MAFA*. Loss of the transcription factors that maintain β-cell identity could result in de-differentiation and hence loss of sentinel β-cell features in the ex vivo human islet cells. A lack of repression of other lineages also promotes a possible transition toward other endocrine or even exocrine cell fates.

**Fig. 1:**
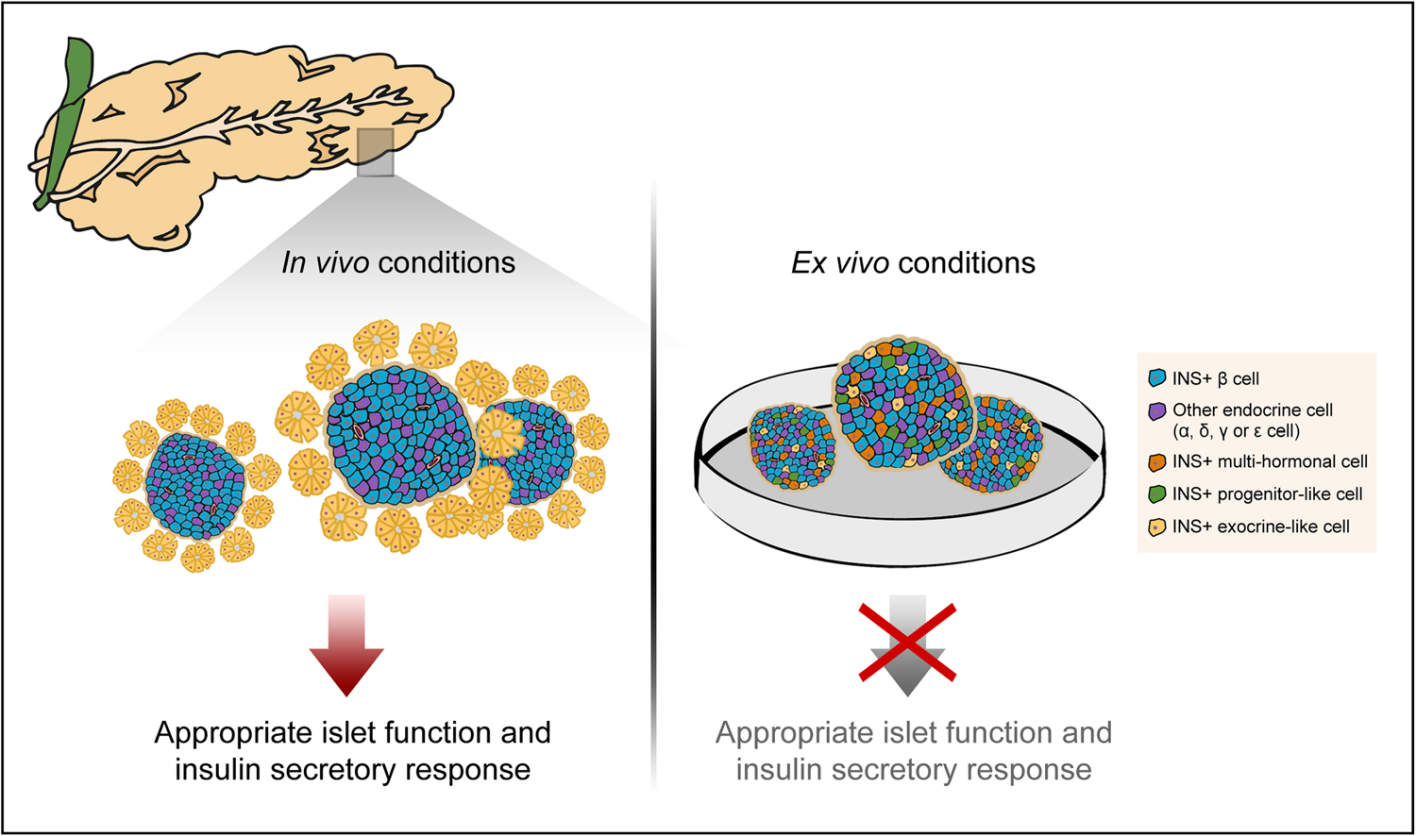
Schematic diagram illustrating the heterogeneity in ex vivo cultured human islets (right) as opposed to the scenario expected in in vivo conditions in the pancreas where INS-secreting β cells (in blue) are predominant (left). The heterogeneity in the isolated islets is characterized by the presence of INS-positive cells that also display expression of multihormonal transcripts, pancreatic progenitor genes, and/or exocrine genes. The de-differentiation signatures observed in these cells suggest that rare pancreatic cells are undergoing cell fate flux, which may have an impact on downstream islet cell function, in particular that of β cells. The population subtypes shown in this diagram are not meant to be mutually exclusive

To gain a better understanding of the identity of these islet cells, the authors zoomed in on their pancreatic transcription factor profile^11^. Strikingly, a large proportion of the cells were found to express pancreatic progenitor markers such as *HNF1B, SOX9*, *NGN3*, and *NEUROD1*, that are not expected of mature islet cells. Many of these single cells also co-expressed pancreatic exocrine genes including *AMY* and *PRSS1*. These results lend support to the de-differentiation signatures observed in the ex vivo human islet cells and consequent expression of multiple hormonal transcripts. While immunostaining analyses have confirmed some of these findings, more extensive work will be needed to confirm the co-expression of transcripts at the protein level and their impact on cellular phenotype and function. The implication of these mixed endocrine, exocrine, and progenitor features is that cells in transitional states are likely to exhibit varying responses to metabolic stimulation and thus contribute to overall islet (dys)function (Fig. 1). Collectively, results from Teo *et al*. suggested that islet cells do not necessarily conform to the markers expected of their cell type, indicating a state of flux at least in ex vivo cultured human islets.

Thus far, all single-cell transcriptomic-based studies on human pancreatic cells remain merely descriptive and largely correlate expression signatures to cellular identity. Current technologies have yet to be able to draw links between expression profile and cellular function at the single-cell level. More sophisticated and innovative methods such as functional assays and imaging techniques that are tailored to single cells and that enable spatial and temporal resolution now need to be employed in future investigations. These will help to establish the biological significance of cellular heterogeneity on a molecular and functional level.

There is accumulating evidence of functional β-cell heterogeneity, although most of these studies have been performed on rodent cells^12^. Functional profiling of individual β cells is crucial in light of a recent report by Johnston *et al*., which revealed that β cells are organized in hubs that are metabolically diverse and likely to contribute to islet insulin release dynamics differently, even if they express high levels of insulin protein^13^. Understanding the specific genomic factors that command these functional responses will be a key question to address. However, one challenge posed by such studies is that single islet cells may not function normally when in isolation, given the lack of necessary cell–cell contacts, autocrine, and paracrine interactions. The study of islet cells in isolation, therefore, may not reflect true single-cell heterogeneity in vivo in the context of an islet and its complex microenvironment^12^. The platforms used for these experiments should therefore be carefully considered and the results need to be interpreted in the context of the limitations of the system.

Single-cell analyses have also been extended to endocrine progenitors and β-like cells differentiated from human embryonic stem cells, to determine the molecular factors that account for heterogeneity during pancreatic endocrine development, at least in vitro^14^. An improved understanding of this complex developmental process that has so far largely been characterized with bulk analyses^15^ will aid efforts to promote maturation/re-differentiation of immature/de-differentiated β cells to restore functional β-cell mass^1^. To this end, single-cell analyses facilitate the characterization of cellular state and cell fate flux within the developing and mature pancreas, and in turn pinpoint specific alterations to this status due to diabetes development. The ultimate goal remains to identify novel therapeutic targets and pathways with potential for tackling diabetes. Finally, the study from Teo *et al*^11^. may have potential implications for the use of human islets for research and clinical transplantation because of the effects of ex vivo culture conditions on islet cell fate and function (Fig. 1). Attempts to reduce cell fate flux ex vivo will be important for applications involving isolated human islets.
